# Comparative Evaluation of Lumpy Skin Disease Virus-Based Live Attenuated Vaccines

**DOI:** 10.3390/vaccines9050473

**Published:** 2021-05-08

**Authors:** Andy Haegeman, Ilse De Leeuw, Laurent Mostin, Willem Van Campe, Laetitia Aerts, Estelle Venter, Eeva Tuppurainen, Claude Saegerman, Kris De Clercq

**Affiliations:** 1 Infectious Diseases in Animals, Exotic and Particular Diseases, Sciensano, Groeselenberg 99, B-1180 Brussels, Belgium; Ilse.DeLeeuw@sciensano.be (I.D.L.); kris.declercq@sciensano.be (K.D.C.); 2 Experimental Center Machelen, Sciensano, Kerklaan 68, B-1830 Machelen, Belgium; Laurent.Mostin@sciensano.be (L.M.); Willem.VanCampe@sciensano.be (W.V.C.); 3 EURL for Diseases Caused by Capripox Viruses, Sciensano, Groeselenberg 99, B-1180 Brussels, Belgium; Laetitia.Aerts@sciensano.be; 4 Department of Veterinary Tropical Diseases, Faculty of Veterinary Science, University of Pretoria, Onderstepoort 0110, South Africa; estelle.venter@jcu.edu.au; 5 College of Public Health, Medical and Veterinary Sciences, Discipline: Veterinary Science, James Cook University, Townsville, QLD 4811, Australia; 6 Institut für Internationale Tiergesundheit/One Health, Friedrich-Loeffler-Institut Federal Research Institute for Animal Health, 17489 Greifswald-Insel Riems, Germany; Eeva.Tuppurainen@fli.de; 7 Fundamental and Applied Research for Animals & Health (FARAH) Center, Research Unit of Epidemiology and Risk Analysis Applied to Veterinary Sciences (UREAR-ULiège), Faculty of Veterinary Medicine, University of Liege, 4000 Liege, Belgium; claude.saegerman@uliege.be

**Keywords:** lumpy skin disease, vaccine evaluation, live attenuated vaccines, lumpy skin disease vaccine

## Abstract

Vaccines form the cornerstone of any control, eradication and preventative strategy and this is no different for lumpy skin disease. However, the usefulness of a vaccine is determined by a multiplicity of factors which include stability, efficiency, safety and ease of use, to name a few. Although the vaccination campaign in the Balkans against lumpy skin disease virus (LSDV) was successful and has been implemented with success in the past in other countries, data of vaccine failure have also been reported. It was therefore the purpose of this study to compare five homologous live attenuated LSDV vaccines (LSDV LAV) in a standardized setting. All five LSDV LAVs studied were able to protect against a challenge with virulent LSDV. Aside from small differences in serological responses, important differences were seen in side effects such as a local reaction and a Neethling response upon vaccination between the analyzed vaccines. These observations can have important implications in the applicability in the field for some of these LSDV LAVs.

## 1. Introduction

Lumpy skin disease virus (LSDV), together with sheeppox virus (SPPV) and goatpox virus GTPV), belongs to the genus *Capripoxvirus* of the family *Poxviridae*. It is a double-stranded DNA virus of approximately 150 kbp [1]. LSDV has a high restrictive host range which limits it to bovini, although tentative links have been made to other Bovidae such as *Oryx* [2]. The observed mortality rates of lumpy skin disease (LSD) are, in general, lower than what is seen for SPPV and GTPV, but exceptions have occurred such as in Oman where a mortality rate of up to 15% was seen [3]. The disease has a severe socio-economic impact due to the fact that livestock is a direct source of income, the reduction in milk yield, weight loss, increased abortion rates and damaged hides [4,5,6] but also provides transport, draught power and fertilizers for agriculture [7,8]. The substantial economic impact [9] and the transboundary nature of LSD warrant the notifiable status of this disease by the OIE and the European Union (Council Directive 82/894/EEC, Commission Decision 89/162/EEC). While LSDV was, for a long time, confined to southern Africa, where it is still endemic, it has spread north- and eastward, affecting the Mediterranean basin in 1988 with outbreaks in Egypt [10] and Israel in 1989 [11]. Continuing its path, LSDV has been reported in the Middle East since the 1990s and the first cases of LSDV in Turkey were reported in 2013 [12]. The virus spread further to the northern part of Cyprus in 2014 and to Greece in 2015 [13]. The emergence into Europe continued with spreading across the Balkan region in 2016 [14]. During that period, LSD also spread to the northern Caucasus and the Russian Federation and was introduced to the Indian subcontinent and China in 2019 and in 2020 to several Southeast Asian countries [15]. 

Vaccination is widely accepted as the most efficient way to control and eradicate LSD. The applicability of a vaccine is determined by its efficiency to prevent the appearance of clinical signs, to block viremia and to reduce virus excretion, resulting in sterile immunity. A vaccine also needs to be safe to use, causing no side effects or vaccinal viremia in vaccinated animals. All these factors are similarly true for the capripoxvirus vaccines. The vaccines currently used and commercially available against LSD are live attenuated vaccines (LAVs). The LAVs used to control and to eradicate LSDV are subdivided according to the strains of viruses used, i.e., LSDV-, SPPV- or GTPV-based. The applicability of LAV, especially LSDV-based, for the control of LSD has, in the past, been demonstrated during the outbreaks in Israel in 2012–2013 [16], in the northern part of Cyprus in 2014–2015 [17] and in the Balkan region in 2016–2017 [18]. However, a relatively small number of studies have been conducted comparing the protection efficiencies of the different commercial LAVs and their safety aspects. This becomes even more important as some studies have reported vaccine failures [19,20,21,22], side effects such as a Neethling response [16,19,23] and the possibility of vaccine virus shedding as demonstrated by the ability to isolate the vaccine virus from nodule-like structures or the detection in vectors and milk [24,25]. Most of the available information is derived from retrospective field studies. Although these investigations often contain valuable data, caution is needed when interpreting and comparing the results on the efficacy and safety of the vaccine because the quality and origin of the used vaccines may not have been demonstrated or the vaccine could be locally produced and not commercially readily available [19,22]. Additionally, the potential previous exposure of vaccinated animals to the field or vaccine virus needs to be taken into consideration, especially when the study is conducted in LSD endemic areas [16,26]. In addition, the age and breed of the cattle population have to be considered as *Bos indicus* breeds in LSDV endemic regions seem to be more resistant to the disease than *Bos taurus* breeds imported from Europe [5,27].

The purpose of this study was therefore to compare and evaluate the efficacy and safety of some commercially available LSDV-based live attenuated vaccines against LSD under standardized conditions.

## 2. Materials and Methods

### 2.1. Challenge Virus and Cell Line

Cells from the ovine testis cell line OA3.Ts (ATCC-CRL-6546) were cultured in DMEM (Fisher Scientific, Merelbeke, Belgium) supplemented with 10% fetal calf serum (FCS; Thermo Fisher Scientific; Belgium), fungizone (1µg/mL; Thermo Fisher Scientific; Merelbeke, Belgium) and gentamycin (20 µg/mL; Fisher Scientific; Belgium).

The LSDV strain LSD/OA3-Ts.MORAN, kindly provided by the Kimron Veterinary Institute, Israel, and the Israeli Veterinary Services, was used as the challenge strain and was propagated on OA3.Ts as described by Babiuk et al. (2007) [28]. Briefly, an 80–90% confluent cell culture flask (175 cm^2^) was inoculated with 200 µL LSDV (10^5^/mL TCID_50_) in 20 mL growth medium (DMEM + 2% FCS containing fungizone 1 µg/mL and gentamycin 20 µg/mL) and subsequently incubated for 4 days at 37 °C in the presence of 5% CO_2_. After a freeze/thaw cycle, the supernatant was collected after centrifugation (3000 rpm, 10 min) and stored in liquid nitrogen as individual aliquots to minimize the effects of freeze/thaw cycles on virus stability.

### 2.2. Serological Analysis

Sera samples were analyzed using the immunoperoxidase monolayer assay (IPMA) and virus neutralization tests (VNT) as described in Haegeman et al. (2020) [29]. Both VNT methods were applied and are referred to as VNT1 (titration of the test serum against 100TCID_50_ of a reference LSDV strain) and VNT2 (titration of an LSDV reference strain against the test serum).

Antibody titrations were carried out based on the results from the IPMA by using a two-fold serial dilution of the test sera. The antibody titer was expressed as the highest sera dilution being positive.

### 2.3. Virus Isolation and Titration

Cell culture flasks (25 cm^2^) were seeded with OA3.Ts and left to grow until 90% confluence was obtained. Culture medium was then removed, and 500 µL EDTA-blood (or homogenized organ/tissue sample) and 500 µL fresh growth medium were added. The cells were incubated for 24 h before 9 mL of additional culture medium was added before a further incubation period of 6 days. The cells were tested for the presence of LSDV using IPMA with some modifications: PBST (PBS containing 0.1% Tween 20) was used for each washing step, and the primary (in-house) positive LSDV serum, secondary anti-bovine IgG-peroxidase antibody and a coloring substrate were applied for visualization.

Virus titration was performed in 96-well plates using an OA3.Ts cell passage with low passage history (passage 4 to 11). Cells were seeded at a concentration of 10^4^ cells/well to achieve 90% confluency after 24-h incubation at 37 °C. The outer wells (columns 1 and 12, rows A and H) of the plate were not used. Plates were infected with 100 µL of a 10-fold serial dilution of LSDV with 10 replicates per dilution and incubated for three days. Viral plaques were detected by IPMA with modification: a positive anti-LSDV serum was used as the primary antibody diluted 1:400 in dilution buffer (1% skim milk powder in 1xPBS). The virus titers were calculated using the method by Reed and Muench (1938) [30], where wells displaying one or more viral plaques were designated as positive.

### 2.4. Animal Trial and Sampling Design

#### 2.4.1. Vaccines

The vaccines included in this study are LSDV-based live attenuated vaccines and are those most frequently used, especially in Africa. All the vaccines were commercially acquired. The following LSD vaccines were included in this study: (1) Lumpy Skin Disease Vaccine (Onderstepoort Biological Products OBP; South-Africa; batch 442); (2) Lumpyvax (MSD-Animal Health; South-Africa; batch BNDM07); (3) Kenyavac (Jordan Bioindustries Center Jovac; Jordan; batch 220115-04); (4) Herbivac LS (Deltamune; South-Africa); (5) Vaccin LSD Neethling O vivant (MCI Santé Animale; Morocco, batch 17BLSDN001). An acronym was allocated to each of the vaccines before the start, allowing anonymous testing in the animal facilities and during the statistical analysis. However, for the purpose of clarity, in this manuscript, the vaccines are referred to as OBP, Lumpyvax, Kenyavac, Herbivac and MCI.

#### 2.4.2. Animal Trial Design

The five vaccines were evaluated during 4 different animal trials in the BSL3 facilities of Sciensano. Each trial had an identical setup and was based upon the efficacy test as described in the OIE Manual Chapter 3.4.12 Section 2.2.4 [31]. All animals were approximately 6-month-old male Holstein bulls which were tested free of BVD and IBR. Upon arrival, all animals were acclimated for 5 to 7 days to reduce the impact of the transport (stress) on the health parameters of the animals [32]. A control group of 5 non-vaccinated animals was included in each trial. Each vaccine group, consisting of 7 animals, was vaccinated according to the manufacturer’s instructions. At 21 days post-vaccination (dpv), the vaccinated as well as the non-vaccinated control animals were challenged with a virulent Israeli LSDV field strain (LSD/OA3-Ts.MORAN; titer 7.5–8 TCID_50_/_mL_ as described in Section 2.3) by intravenous (5 mL, vena jugularis) and intradermal (1 mL) inoculation. The intradermal injection was performed at 2 locations on both sides of the neck (250 µL per site). After the challenge, the animals were monitored and sampled for at least 21 days. 

All animal experiments were conducted according to the European Union and Belgian regulations on animal welfare in experimentation. The protocol was approved by the joint Ethical Committee of Sciensano, authorization number 20150605-01_EC_Dierproef aanvraag_LSDV_BMG_2015.

#### 2.4.3. Clinical Evaluation and Scoring

During the complete duration of the animal trial (acclimatization, post-vaccination and post-infection), all animals were daily clinically evaluated. These daily observations were translated into points, using Table 1, and applied for calculating an overall total clinical score. In addition to the parameters listed in Table 1, the animals were also checked for a local reaction at the vaccination site, conjunctivitis, nasal discharge and diarrhea.

Following vaccination, lump-like structures were sometimes observed on the animals. These nodules are similar to those seen after challenge but are clearly smaller. These nodules developed into small skin lesions with scab formation but complete healing was seen in 1 to 2 weeks. This type of post-vaccination reaction is here referred to as a Neethling response.

Following challenge, two types of nodules were observed: (1) lump-like structures which developed during the course of the experiment into skin lesions with scab formation and remained visible until necropsy—this type of nodule is referred to as a permanent nodule; (2) lump-like structures which are indistinguishable from the permanent nodules but disappear after a few days without the formation of skin lesions or scabs—this type of nodule is referred to as a temporary nodule.

#### 2.4.4. Sampling

Samples for laboratory evaluation were taken as follows: (1) once during the acclimatization period (between 3 and 5 days prior to vaccination); (2) on the day of vaccination but before the injection (0 dpv); (3) 3 times a week during the post-vaccination period; (4) on the day of challenge but before the injection (0 dpi); (5) on a daily basis from 5 to 15 dpi and every other day before and after this period. These include: EDTA blood, serum and heparin blood. Biopsies were taken when nodules first appeared to confirm the presence of LSDV. At necropsy, approximately 25/26 tissue and organs samples were collected per animal (cfr. Table 2 (*n* = 25); for the vaccinated animals, the site of vaccination was taken as well). When animals had developed nodules, a part of the skin with no nodules in the vicinity (more than 15 cm) was taken and is referred to as “normal skin”.

### 2.5. Real-Time PCR (RT-PCR)

#### 2.5.1. Pan Capripox RT-PCR

The lumpy skin disease virus genome was detected using the Pan Capripox real-time PCR panel, consisting of three real-time PCRs (D5r, E3L and J6R), described by Haegeman et al. (2013) [33]. The panel was used as follows: after an initial screening with the D5R real-time PCR, samples were tested with the E3L and J6R real-time PCRs if: (a) the Cp > 37 or (b) in a time consecutive sample series, the status of the animal changed (negative to positive and vice versa).

#### 2.5.2. DIVA Real-Time PCR (DIVA RT-PCR)

Differentiation between the LSDV vaccine and wild-type LSDV genome was performed using the real-time DIVA PCR described by Agianniotaki et al. (2017) [34].

### 2.6. IFNg Release Assay

The secretion of interferon gamma, following the stimulation of heparinized blood, was examined using the BOVIGAM^®^ 2G kit (Thermofisher; Merelbeke, Belgium). The methodology was based upon the manufacturer’s instructions and Parida et al. (2006) [35] and consisted of a stimulation stage and the detection of IFNg with a sandwich ELISA. Heparinized blood samples were analyzed within 24 h after collection. The heparin tubes were gently inverted before blood was transferred to a 24-well plate. Each sample was placed in triplicate on the plate with each well containing 1.5 mL of blood. This allowed each sample to be stimulated with: (a) 1× PBS (100 µL/well), as a negative control; (b) pokeweed mitogen (100 µL/well at 160 µg/mL dissolved in 1x PBS), as a positive control; and (c) LSDV strain LSD/OA3-Ts.MORAN (100 µL/well at 6.8 TCID_50_/_mL_). The stimulating agent was mixed with the blood by pipetting, and the 24-well plate was then incubated overnight at 37 °C. The plasma was collected after centrifugation at 500× *g* for 10 min at room temperature and stored at −20 °C until analysis. The cut-off of the sandwich ELISA for positivity was set to 0.3. The OD values of the positive and negative controls were monitored over time to identify false positive and negative results. A response was classified as strong, medium and weak when the corrected OD (=OD virus–OD PBS) was >2; between 1 and 2; and <1, respectively.

### 2.7. Statistical Analysis

A two-factor ANOVA (Anova 2D) with repeated measures on one factor (the body temperature, the swelling size and the clinical scoring) was used to compare the kinetic of different parameters between groups of animals [36]. The validity conditions (homogeneity of variances and covariance matrixes) were preliminary tested [37]. The difference in the peak of temperature between the different vaccinated groups was tested using a Kruskal–Wallis equality-of-populations rank test. For DNA detection in organs/tissues using the PCR test, and for VNT, INFg and IPMA detection, frequencies and proportions were compared with the Pearson chi-square test or Fisher’s exact test (in the case of a small effective sample size) for count data [36]. The difference in the PCR Cp value between the positive samples from clinical and non-clinical animals was tested using the two-sample Wilcoxon rank-sum (Mann–Whitney) test [36]. Association between the number animals that were positive for VNT or INFg for the different vaccinated groups at the post-vaccination period, at challenge and at the end of the trials was tested using a non-parametric Spearman correlation coefficient. For all tests, *p*-values < 0.05 were considered significant.

## 3. Results

### 3.1. Unvaccinated Infected Control Group: Clinical Observations and Scoring

**Figure 1:**
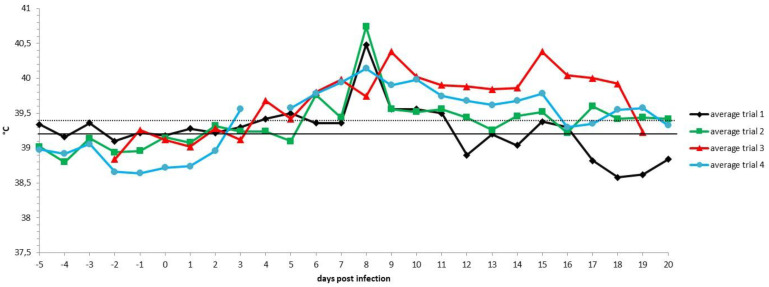
Averaged body temperatures per trial of the control animals. Solid line: fever cut-off for consecutive days; dotted line: fever cut-off for isolated fever days.

Following the inoculation with a virulent LSDV, all animals in the control groups (*n* = 20) developed a fever which spiked around 7 to 9 dpi. The highest body temperature measured was 41.4 °C, with 90% and 35% of the animals having a maximum body temperature above or equal to 40 °C and 41 °C, respectively. Following the fever spike, the body temperature returned relatively quickly (1 to 6 days) to normal in 55% of the control animals. In contrast, in 45% of the animals (9/20), the fever or elevated body temperatures remained for a prolonged period of time (>10 days). The body temperature patterns were similar across the four trials (Figure 1). The impact of infection on the feeding behavior was very limited with only two animals (10%) showing a reduced feed uptake at 7 dpi. One of those animals, con2_trial4, stopped eating at 14 dpi. This animal was also the sole control animal that showed a light (7 dpi) to severe (14 dpi) change in its general health status and was therefore euthanized due to ethical reasons at 15 dpi. Enlarged prescapular lymph nodes were observed in 25% of the animals (5/20) starting from 7 to 8 dpi onwards. The enlargement coincided with the observed fever spike. The prescapular lymph nodes remained enlarged until the end of the trial and all five animals developed skin nodules. Nodules appeared in 10 out of 20 animals (50%) between 6 and 10 dpi and remained visible until the end of the trial. The appearance of the permanent nodules began either localized (on one or two places on the animals, 80%) followed by a generalization after 1 to 7 days or was, from the beginning, multifocal on several places on the animal. There was only one exception, namely, con2_trial1, where the nodules disappeared after five days. Interestingly, eight out of the nine animals with permanent skin lesions had a prolonged fever, while this was observed only in one animal (10%) of the group, showing no skin lesions at all.

**Figure 2:**
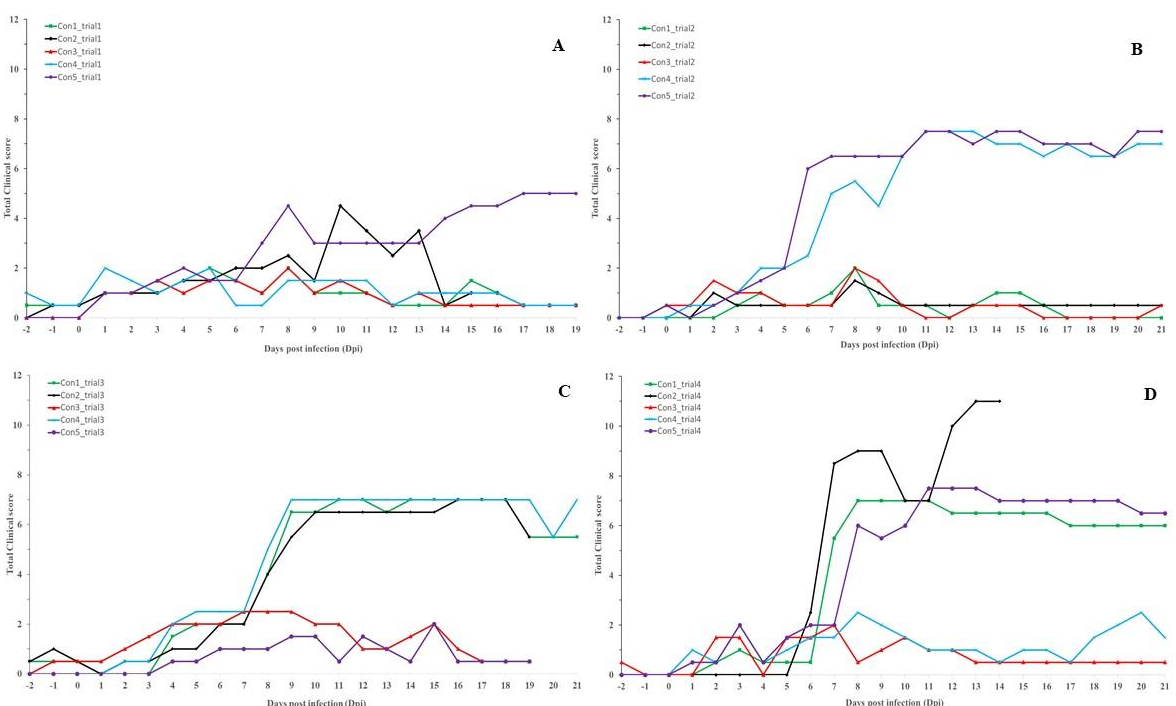
Total clinical score of the control animals. Infected at 0 dpi; (**A**): Trial 1; (**B**): Trial 2; (**C**): Trial 3; (**D**): Trial 4.

The difference in the clinical picture within the control group became visible between 6 and 8 dpi (Figure 2). During this period, the total clinical score of all the animals which developed permanent nodules and viremia surpassed a clinical score of 3 and continued to rise afterward. For animals that did not develop nodules or viremia, in general, the total clinical score did not surpass 2 with two exceptions, namely, con2_trial1 and con4_trial4. These were due to the appearance of temporary nodules (con2_trial1) and a 3-day fever period (con4_trial4) at the end of the trial.

**Figure 3:**
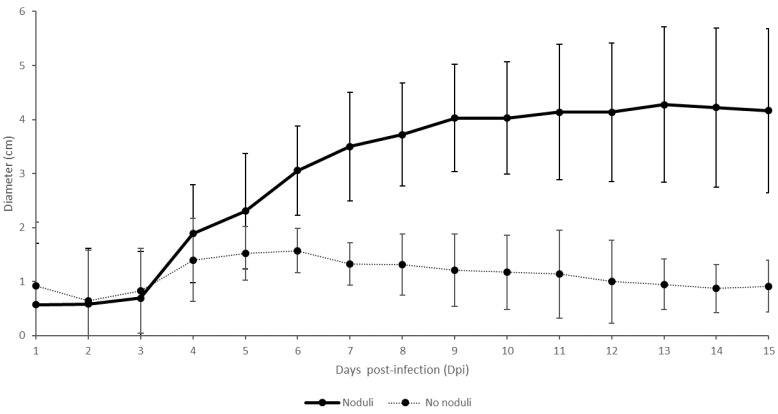
Averaged swellings at the virulent virus inoculation sites (intradermal) on animals with and without skin nodules (in cm). Variation is shown by the error bars.

A small swelling was seen at the four sites of intradermal injection after the first day (1 dpi) on all animals. In 30% of the animals, the swellings remained small (<2 cm) and, in some cases, healed before the end of the trial. None of these animals developed nodules or became viremic. The swellings at the inoculation site on the remaining animals continued to grow and achieved moderate status between 2 and 6 dpi. On six animals (30% of total), the swellings at the inoculation site of the virulent virus increased further to reach large or very large status. All these animals developed skin nodules and became viremic. The swellings on the remaining eight animals remained either moderate until the end of the trial (*n* = 3) or started to decrease in size and became small again (*n* = 5) after a few days. Viremia and formation of skin lesions were only seen in the three animals where the swellings remained moderate (100%). In the other five animals, only animal Con2_trial1 developed temporary nodules, but viremia was never seen in these five animals. The difference in the size of the local swelling between the animals with and without skin lesions is, in general, seen around 5 dpi (Figure 3). A two-factor ANOVA with repeated measures on one factor was used to compare the swelling size on animals with and without nodules and a significant difference (starting from 7 dpi) was observed between the two groups of animals over time (*p*-value = 0.01).

### 3.2. Unvaccinated Infected Control Group: Virology and Serology

**Figure 4:**
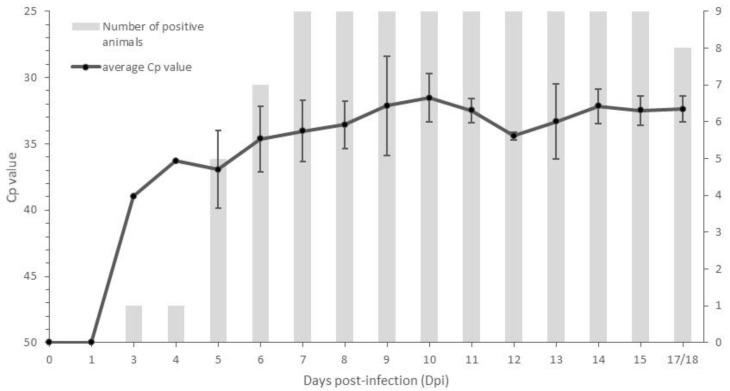
Control animals displaying skin nodules (*n* = 9): average Cp of the positive blood samples (standard deviation is shown as error bars) and the number of animals with a PCR-positive status.

Viremia was detected by PCR in 100% of the animals which had skin lesions (*n* = 9), while no viremia was detected in animals without any skin nodules (*n* = 10) and the animal with the temporary nodules (*n* = 1). The onset of viremia varied between 3 and 7 dpi with the majority of onsets occurring at 5 dpi (*n* = 4). Viremia remained relatively stable with Cp values between 32.5 and 36 until 17 to 18 dpi (Figure 4) with only one animal becoming PCR-negative closer to the end of the trial (17 dpi). At the end of the trials, approximately 25 samples were taken from different organs and tissues from 15 control animals (6 which had developed skin lesions/viremia and 9 without). The percentage of PCR positivity for each sample type in animals with or without skin nodules or viremia is summarized in Table 2. Within each trial, the number of PCR-positive organs/tissues was clearly higher (two to six times) in animals that had developed nodules/viremia compared to animals that had not. Considering the same pattern for each of the organs/tissues, the sum of the positive PCR results was compared between the two groups of animals. The percentage of positive organs/tissues was significantly more important in the group with nodules/viremia (Pearson chi2 (1 degree of freedom) = 106; *p*-value < 0.001). In the animals showing skin nodules and viremia, a 100% PCR detection rate was seen in skin lesions and also in three out of the four collected muscle types. Additionally, lymphoid tissues, such as parotid and lymph nodes (inguinal, mesenterial, submandibular, prescapular lymph nodes), had a high degree of positivity (83 to 100%). Aside from the difference in the number of positive samples, a significant difference in the PCR Cp value between the positive samples from clinical and non-clinical animals could be observed (Mann–Whitney test; <0.05). In general, this difference was between 4 and 6 Cps but could be as high as 16 (nasal mucosa: Cp 22.6 vs. 38.6 with *p*-value = 0.002 using the Mann–Whitney test; the inoculation site of the challenge virus: Cp18.1 vs. 34.5 with *p*-value = 0.045 using the Mann–Whitney test). Interestingly, a high percentage of LSDV DNA was detected in normal skin type samples (66.7%) in animals which had not developed nodules or viremia. LSDV DNA was only sporadically detected in these animals and also in other organs/tissues with the exception being the site of challenge virus inoculation (88.9%).

**Figure 5:**
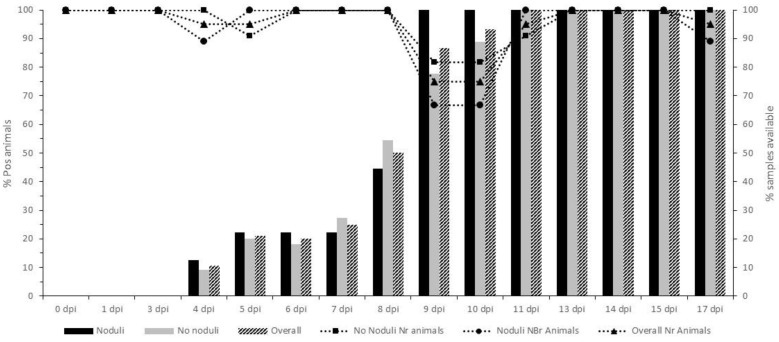
Seroconversion of the control group determined by IPMA. The number of seroconverted animals (in percentage) is displayed in bars, while the percentage of animals sampled is depicted as lines.

The onset of seroconversion was determined at 4 dpi using IPMA, and at 13 dpi, all animals had seroconverted and would remain so until the end of the trial (Figure 5). No difference in seroconversion was seen between animals with and without nodules. Neutralizing antibodies were, for the first time, detected, using VNT1, between 9 and 15 dpi (average 12.4 dpi) for the clinical group and between 15 and 17 dpi (average 15.5 dpi) in the non-clinical animals. The difference in onset detection with the IPMA was, on average, 4 and 6.7 days for the clinical and non-clinical groups, respectively. Comparable data were obtained with VNT2, where the onset was between 11 and 15 dpi for the clinical group (average 13 dpi) and between 11 and 17 dpi (average 14 dpi) for the non-clinical animals. The difference between the IPMA and the VNT2 was, on average, 4.6 and 5.4 days. Although seroconversion, as detected by VNT1 and VNT2, was 100% in the clinical group from 15 dpi onwards, this was not the case for the non-clinical group. In addition to the three animals which remained negative in VNT1 and VNT2, two animals seroconverted only in VNT2 and one only in VNT1. The average antibody titer at the end of the trial (20/22 dpi) was 1/16457 and 1/4058 for the clinical and non-clinical groups, respectively.

### 3.3. Vaccinated Groups: Clinical Observations and Scoring

#### 3.3.1. Post-Vaccination

**Figure 6:**
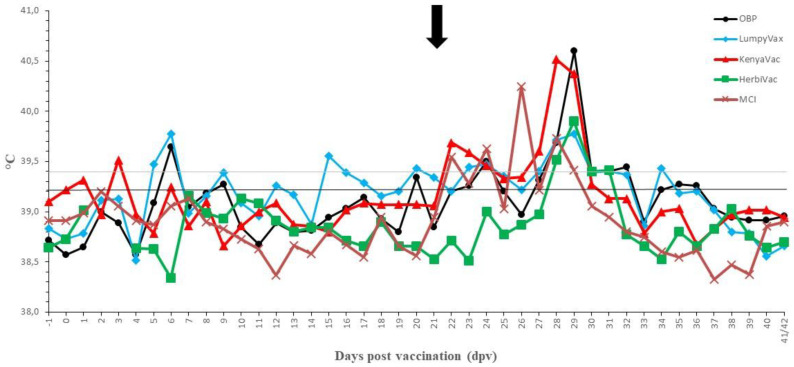
Averaged body temperatures of the vaccinated groups. Solid line: fever cut-off for consecutive days; dotted line: fever cut-off for isolated fever days; arrow: day of challenge.

Although a raise in body temperature was seen in all groups, upon vaccination, the extent of this fever response differed significantly between the vaccines, as can be seen in Table 3 (Anova 2D, *p*-value = 0.01). For all vaccines, the onset of elevated body temperature was relatively quick after vaccination (sometimes at 1 dpv) and the peak was mostly seen between 6 and 9 dpv. No significant difference in occurrence of this peak could be demonstrated between the vaccinated groups (Kruskal–Wallis equality-of-populations rank test; *p*-value = 0.12). However, for Lumpyvax, the fever profile was intermittent with a second peak around 15–17 dpv. The average temperature profile for all vaccines is depicted in Figure 6.

**Figure 7:**
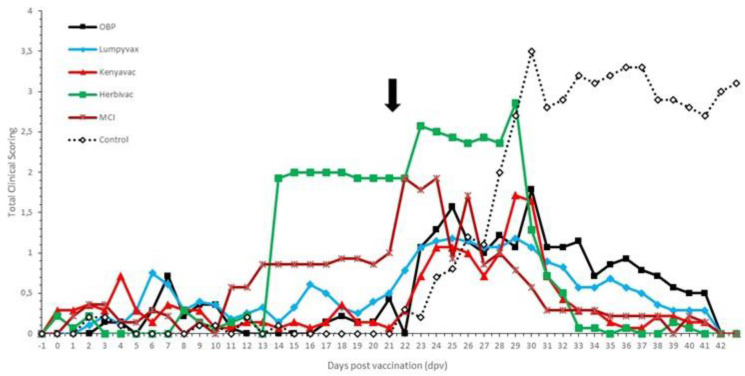
Averaged total clinical score of the vaccinated groups. For comparison purposes, the average of the control groups was added (dotted line). The black arrow indicates the day of challenge.

No negative impact of the vaccination was seen on the feed uptake, general behavior and general health status in all groups. The sole exception was the enlargement of the prescapular lymph nodes in the Herbivac group. No clear local reactions were seen at the site of vaccination except in the MCI group where three animals (43%) had a severe swelling (>10 cm diameter). This swelling remained visible throughout the animal trial. No evidence was found for the development of a Neethling response in the OBP, Lumpyvax and Kenyavac groups. However, animals in the Herbivac (*n* = 3) and MCI (*n* = 2) groups developed small nodule-like structures on 11 dpv (MCI) and 13 dpv (Herbivac). These nodules remained small, did not evolve any further in contrast to nodules on infected animals and remained visible until 24 dpv and 28 dpv, respectively. A sample was taken from the nodule-like structures from all animals. The presence of LSDV was confirmed with the Pan Capripox RT-PCR and identified as vaccine LSDV with the DIVA RT-PCR. The Neethling response in the Herbivac group coincided with the enlargement of the prescapular lymph nodes, while this was not the case for the MCI vaccine. All parameters were scored and averaged per vaccine and are summarized in Figure 7. A significant difference could be seen post-vaccination in the clinical scoring over time between the OBP, Lumpyvax and Kenyavac vaccine groups, on the one hand, and the Herbivac and MCI groups, on the other hand (Anova 2D; *p*-value = 0.01).

#### 3.3.2. Post-Challenge with a Virulent Field LSDV Strain

Except for the Herbivac group, a small raise in body temperatures was seen around 1 to 3 dpi in the majority of the animals. As this is shortly after challenge, the impact of stress cannot be ruled out. A clear fever spike (>40 °C) was seen between 5 and 8 dpi in 100% (Kenyavac, Herbivac, MCI), 86% (OBP) and 71% (Lumpyvax) of the animals. For the Kenyavac-, Herbivac- and MCI-vaccinated animals, the body temperature returned quickly to normal (10 to 11 dpi). Although the body temperatures in the OBP- and Lumpyvax-vaccinated animals also declined quickly, they remained slightly raised (39.4–39.7 °C) until 15/16 dpi. Aside from the fever spike, all animals were protected against other clinical signs, including the typical LSD nodule formation. The number of days with elevated clinical scoring post-challenge of the vaccinated animals was significantly lower than that of the control animals (Anova 2D; *p*-value = 0.01). All parameters were scored and averaged per vaccine and are summarized in Figure 7. The local reaction at the sites of intradermal injection was very similar for all vaccines tested. After an initial swelling of 1 to 2 cm in diameter, the reaction started to dissipate between 4 and 5 dpi and was barely visible (<0.5 cm) or completely healed from 7/8 dpi onwards. A two-factor ANOVA with repeated measures on one factor showed a significant difference in the swelling size over time (*p*-value = 0.01), starting from 7 dpi, between vaccinated animals and control animals with skin lesions.

### 3.4. Vaccinated Groups: Virology

No viremia, related to the vaccine or challenge virus, could be demonstrated in the OBP, Lumpyvax and Kenyavac groups, although isolated positive Pan RT-PCR results were obtained for Lumpyvax on 6 dpv (*n* = 2) and 8 dpi (*n* = 1) and for Kenyavac on 13 dpv (*n* = 1). As they were shortly after vaccination or infection and had high Cp values (>38), it is more likely that they were caused by the injected vaccine/challenge virus rather than true viremia. For Herbivac, however, vaccine-related viremia was detected in two animals, of which only one animal developed nodules (Neethling response). This viremia occurred between 11 and 15 dpv, with a viremic period of 3 to 5 days. In addition, two other animals, which had a Neethling response, were found PCR-positive on 11 dpv only. Using the DIVA RT-PCR, the virus present could be typed as vaccine LSDV in three of the four animals. In the MCI group, only one of the two animals with a Neethling response was PCR-positive and only for one sampling at 9 dpv (confirmed as vaccine LSDV). A second animal, without a Neethling response, was PCR-positive between 3 and 7 dpv, albeit with high Cp values (>38). For both vaccines (Herbivac and MCI), no virus could be detected in the blood after challenge.

Similarly to the control group, several organs/tissues were collected at necropsy and tested for the presence of the challenge or vaccine virus. The number of positive samples was very similar for OBP (*n* = 11; 7.9%), Lumpyvax (*n* = 7; 5%), Kenyavac (*n* = 10; 7.1%) and MCI (*n* = 7; 5%) vaccines. A slightly higher number of positive organ/tissue samples (*n* = 26 or 19.7%) was obtained for the Herbivac vaccine. No specific pattern could be observed in regard to sample positivity and the obtained Cp values were higher than 36 (except one normal skin sample of the Herbivac group which had a Cp of 32). The sample type with the highest degree of positivity was observed for the inoculation site of the challenge virus. For OBP, Lumpyvax, Kenyavac and Herbivac, the inoculation site was positive in 43% of the cases with a Cp value of 36/37 for OBP/Kenyavac and 31/32 for Lumpyvax/Herbivac. In contrast, no virus could be detected for this sample in the MCI group. Additionally, “normal” skin samples were found positive in 71% (5 of 7) of the animals vaccinated with Herbivac and in 43% (3 of 7) of the Kenyavac-vaccinated animals, while this was only 14% (1 of 7) for OBP and Lumpyvax and 0% for MCI. The observed difference between Herbivac/Kenyavac and OBP/Lumpyvax/MCI was found to be statistically significant (Fisher’s exact test; *p*-value = 0.013).

### 3.5. Vaccinated Groups: Serology

**Figure 8:**
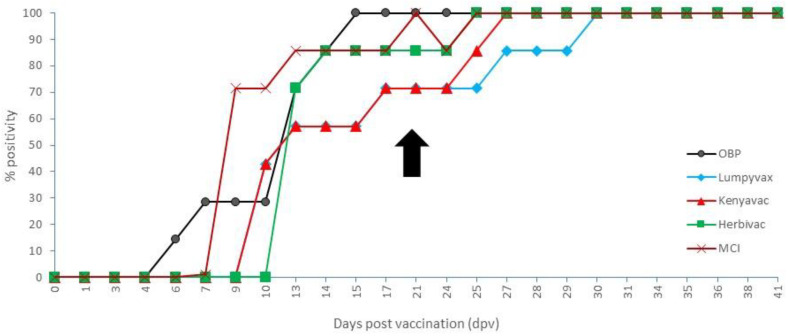
IPMA results of the vaccinated animals. Black arrow: day of challenge.

The onset, as demonstrated by the IPMA, varied among the different vaccines between 6 (OBP) and 13 dpv (Herbivac). Complete seroconversion was seen between 15 (OBP) and 29 dpv (Lumpyvax). When looking at the day of challenge (21 dpv), the seroconversion rate was 100%, 71.5%, 71.5%, 86% and 100% for OBP, Lumpyvax, Kenyavac, Herbivac and MCI, respectively, but no statistical difference between the vaccine groups was found (Fisher’s exact test; *p*-value = 0.50). All animals remained positive until the end of the trial. The IPMA results are summarized in Figure 8. The antibody titers at the time of challenge were similar for OBP, Lumpyvax and Kenyavac and averaged between 1/550 (OBP) and 1/1080 (Kenyavac), whereby the highest individual titers were found for Kenyavac. Somewhat higher averaged titers were found for MCI and Herbivac vaccines (average 1/1600 and 1/2240), although the highest individual titer was similar for both (1/3840). A clear booster effect on the titers was seen following challenge for all except the Herbivac group. It was most pronounced for Lumpyvax, where the averaged titer at the end of the trial was 1/4000 (7x increase), while for the other three vaccines, the increase was two- to three-fold. For Herbivac, the averaged titer remained identical, as did the minimal and maximal individual titers (1/960 and 1/3840).

The onset of neutralizing antibodies when using VNT1 was found between 13 (Lumpyvax) and 24 dpv (3 dpi; Kenyavac). No complete seroconversion was seen within any of the vaccine groups during the duration of the trial. At the moment of challenge, a seroconversion rate was seen of 14.3% (OBP), 14.3% (Lumpyvax), 0% (Kenyavac), 42.9% (Herbivac) and 57.2% (MCI). The number of animals that seroconverted was significantly less in the groups vaccinated with OBP, Lumpyvac and Kenyavac in comparison with the groups vaccinated with Herbivac and MCI (Fisher’s exact test; *p*-value = 0.015). A booster effect was seen in all groups except the MCI group as the seroconversion rate at the end of the trial was 42.9% (OBP), 71.4% (Lumpyvax), 42.9% (Kenyavac), 85.7% (Herbivac) and 57.2% (MCI). The number of animals that seroconverted at the end of the trial was not significantly different between these groups of vaccinated animals (Fisher’s exact test; *p*-value = 0.49). However, a number of animals in the OBP, Lumpyvax and Kenyavac group fluctuated around the cut-off at the end of the trial, meaning that only two to three (28.6 to 42.9%) animals in these three groups were still clearly antibody-positive at the end. Averaged neutralizing titers were similar (1/200 to 1/375) among the vaccines except for MCI-vaccinated animals which had a slightly higher averaged titer (1/700). No association was evidenced between the number of animals that seroconverted for the different vaccinated groups at challenge and at the end of the trials (Spearman correlation coefficient = 0.55, with *p*-value = 0.33).

The onset of neutralizing antibodies as detected by VNT2 was not unlike the one seen with VNT1, namely, between 10 (Lumpyvax) and 17 dpv (Kenyavac). However, the seroconversion rate at the time of challenge was higher (42.9%, 57.1%, 28.6%, 71.4% and 42.9%), but the observed differences between these groups of vaccinated animals were not significant (Fisher’s exact test; *p*-value = 0.71). Similar to VNT1, no complete seroconversion was observed with VNT2 in any of the vaccine groups during the trial. At the end of the trial, neutralizing antibodies were detected in 71.4 % (OBP), 85.7 % (Lumpyvax), 57.14% (Kenyavac), 85.7% (Herbivac) and 57.14% (MCI) of the animals, but the difference between the groups was not significant (Fisher’s exact test; *p*-value = 0.73). The respective averaged neutralizing index (NI) was very similar among all groups, varying between (1.9 and 2.1).

### 3.6. Vaccinated Groups: Cellular Immunity (IFNg Assay)

To evaluate the cellular immune response after vaccination, IFNg secretion was measured after stimulation of the heparinized blood with the LSDV antigen. In the post-vaccination period, 100% of the vaccinated animals in the Kenyavac group displayed an IFNg response whereby the great majority of the animals (71%) reacted strongly. High IFNg responsiveness (86% of the animals) was similarly found in the Herbivac, Lumpyvax and MCI groups with 86%, 57% and 57% responding strongly, respectively (Supplemental Figure S1). In the OBP group, a significantly lower number of animals showed an IFNg response compared to the other vaccinated groups (57%; Fischer’s exact test; *p*-value = 0.04), of which 43% reacted strongly. Although a decrease over time was seen in all groups, this was most pronounced for Lumpyvax and Kenyavac. At the time of challenge, only 28.6% (Lumpyvax) and 57% (Kenyavac) of animals were still responsive. In contrast, this decrease in responsiveness was less pronounced for OBP and Herbivac with 43% and 86% of the animals still reacting at 21 dpv. This represents a decrease of only 14% or one animal. This limited decrease in responsiveness is even more true for Herbivac, as still 57% of the animals reacted strongly, while only weak responders were found for OBP at this time point. MCI was positioned in the middle with still 57% of responders, which is a decrease of 29%. No association was evidenced between the number of responders for the different vaccinated groups at the post-vaccination period and the time of the challenge (Spearman correlation coefficient = 0.70, with *p*-value = 0.19).

## 4. Discussion

Five commercially available live attenuated LSDV-based vaccines were included in this study. Although the Kenyavac vaccine is advertised by the manufacturer as a live sheeppox and goatpox strain KSGP0240 vaccine, sequence data from the RPO30 and GPCR genes strongly suggested that this vaccine strain is LSDV-based [38]. This was confirmed by the complete sequence of the vaccine strain by Vandenbussche et al. (2016) [39] and was therefore selected for this study. The fact that it can be readily differentiated from SPPV and GTPV with a species-specific real-time PCR [38] casts doubts about the background and quality checking of this vaccine.

Due to the number of animals involved and the resulting practical implications to evaluate five different vaccines, the evaluation was spread over multiple animal trials. However, all the animals originated from the same source and were of a similar breed and age, ensuring homogeneity of the used bovine population. Very little variation was seen in the clinical, virological and serological parameters between the four control groups. Two to three animals in each of the four control groups developed the typical LSDV skin nodules with the onset between 6 and 8 dpi. All animals developed fever with the highest temperatures measured between 7 and 9 dpi. These data are comparable to previously published data [22,40,41,42,43]. Clear/detectable viremia (on multiple consecutive days) was only detected in the animals which developed nodules with the onset between 3 and 7 dpi. These results corroborate results by Möller et al. (2019) [42] from a challenge study using the LSDV Macedonian strain. All control animals seroconverted before 13 dpi. Based upon similar findings during different animal trials conducted during the study and previously published data, it can be concluded that the challenge in all trials was successful and that this challenge model is robust and can be used to compare the different vaccines. Virus distribution at necropsy was significantly more pronounced (two- to four-fold) in control animals showing clinical signs compared to asymptomatic animals within each of the animal trials. This is indicative of an association between clinical signs and virus multiplication with systemic spread. The viral load in the samples collected from animals showing no clinical signs was, on average, 3 and 5 Cps lower, but the difference could go as high as 16 Cps (nasal mucosa). The presence of LSDV in “healthy parts” of the skin has been reported previously but these samples came from animals which did have nodules on other parts of the skin [17]. In the presented study, a high number of normal or “healthy” skin samples (66.7%) were positive from infected animals without any skin lesions or viremia. This is an interesting finding in view of future screening procedures. The exact epidemiological implications are currently unclear and warrant further study. Transmission from “healthy” looking skin by means of ticks has been described, but this was from viremic animals which the animals in this study were not [44].

In general, the vaccines were well tolerated by all the animals as the impact of vaccination on the feed uptake, behavior and general health status of vaccinated cattle was almost nonexistent. Very limited local reaction at the site of vaccination was seen in all the vaccine groups except for the MCI group. Skin reactions due to vaccination were reported previously. A study in Greece reported 12% of the animals vaccinated with OBP developed a local reaction [23]. It needs to be kept in mind that in the study from Greece, the target populations were dairy cattle with a greater diversity in sex and age than the animal population in this study. Furthermore, when only the calves between 3 and 12 months old are considered from the Greece study, only 2% developed a reaction. Keeping in mind the limited number of animals per vaccine group, our data support this very low percentage with the exception of the MCI group. For the MCI group, the percentage is higher than the others and although a small local reaction at the vaccination site is sometimes considered as a proof of pox vaccine immunogenicity, it can pose problems for its application in the field in Europe and elsewhere. This level of local reaction was not seen in Bulgaria when the MCI vaccine was applied in a cattle population that was already vaccinated with the OBP or Lumpyvax vaccine for 3 consecutive years (Tsviatko Alexandrov, personal communication). This is in agreement with previous reports that stated the most severe LSDV vaccine side effects are only seen when cattle are vaccinated for the first time with live attenuated LSD vaccines [45].

Following vaccination, a fever response was often observed in the vaccinated animals. The extent (duration, number of animals and severity) varied between the groups. It was most pronounced for Lumpyvax while less so in MCI- and Herbivac-vaccinated animals. Interestingly, the timing of the fever spikes is similar to that seen after infection, namely, around 6/7 days after application. This is most probably the result of the replication of the LAV virus inducing the immune response. The finding that the fever response was less in Herbivac and MCI was surprising as a Neethling response was seen in 43% (Herbivac) and 28% (MCI) of the vaccinated animals. A Neethling response has been previously reported for OBP and Lumpyvax at very low percentages and varied between less than 0.1% [46] and 0.38% [16], sometimes reaching 1.5% [24]. Our data for OBP, Lumpyvax and Kenyavac fall within these observations. In contrast, a higher prevalence of 9% was reported for OBP by Katsoulos et al. (2018) [23] in dairy cattle in Greece. The difference in prevalence may be associated with a low number of experimental cattle used in our study, their age and stage of production. In view of the Neethling response detected in the Herbivac and MCI groups, the finding of vaccine viremia in our study is not surprising. More interesting is the finding of vaccine viremia in some animals of both above-mentioned groups that did not develop any nodules after vaccination. This subclinical form of a Neethling response was also found by Bedekovic et al. (2018) [24] with the OBP and Lumpyvax vaccines. In our study, we did not observe this for the OBP and Lumpyvax vaccines, but this is probably due to the limited number of animals per group. The epidemiological importance of this finding in a field setting needs further study.

Antibodies were earlier detected in the vaccinated animals using IPMA compared to the VNTs, which is in agreement with our previous findings [29]. A high seroconversion rate was seen with the IPMA, ranging from 71% (Lumpyvax) to 100% (OBP and MCI) at the time of challenge, while this was certainly not the case when using VNTs (0% to 71%). This is probably because only neutralizing antibodies are measured with the VNT. Protection in the absence of neutralizing antibodies was also reported by Osuagwuh et al. (2007) [47]. This can also be seen for the seroconversion strength at the time of challenge, whereby Herbivac had higher titers on IPMA while being similar to the others on both VNTs. All these elements have to be kept in mind when making conclusions about the protective capacity. Nevertheless, when also the IFNg results are considered, it can be stated that the immunological response was strongest in the Herbivac group followed by MCI and then the three other vaccines. It can be questioned if this higher immunogenicity, and increased Neethling response, for Herbivac can be linked to the restoration of certain gene functions such as B22 [48] or an SOD homolog [49] in this vaccine strain. This becomes even more relevant as an SOD homolog has been tentatively linked to enhanced immunogenicity [50].

Aside from a fever response, which is slightly more pronounced in OBP and Lumpyvax, all animals were protected against a virulent LSDV challenge. This is demonstrated by the significant difference between the clinical score of the vaccinated and the control animals. The timing of the fever is reminiscent of the one seen in the control group, albeit more reduced in time. No other clinical signs were seen and no LSDV viremia was found in all vaccinated animals. The latter combined with the fact that, at necropsy, only traces (high Cp values of >38) of the LSDV genome were found in a limited number of organs and tissues confirms the equal protection conferred by all vaccines. No statistical difference was found at the serological and cellular immunological (IFNg responsiveness) levels with the exception of VNT1 post-vaccination. This significant difference in neutralizing antibodies between the vaccine groups had no influence on the clinical protective capacity of the vaccines used. Most probably, all vaccinated animals exceeded the minimum immunity level needed for protection and therefore no difference was seen among the vaccines.

Looking at the experimental setup, two limitations can be identified. Firstly, the number of animals that could be included was limited to seven (vaccine groups) or five (control groups). Aside from the practical and ethical difficulties in conducting animal experiments with large farm animals in BSL3 facilities, the number of animals used during this study in the different groups (vaccine and control) is in line with the guidelines of the OIE Terrestrial Manual for efficacy studies [31]. Furthermore, several of the observations/findings, most importantly the efficacy of all included live attenuated vaccines against a viral LSDV challenge, could be validated statistically, indicating that the number used was sufficient for the purpose of this study. Secondly, the animals used in the different trials were all males. However, LSDV affects both sexes and the severe form has been found in males as well as in females [51,52]. Looking for differences in the susceptibility between males and females is confounded by the impact of breed, age and production system which makes it difficult to pinpoint the true effector. However, no statistical difference was found between males and females in several studies [53,54,55,56]. A higher seropositivity was reported in female animals in Uganda [57], but this could be due to fact that female animals are kept for longer by the farmer than males, which are quickly sold off. Therefore, the observed difference could be linked to the duration of exposure rather than an effect of the sex of the animal. Lactating cows have been reported to be more susceptible to LSDV [52,54]. Although the exact impact on the presented data is difficult to predict, an increased susceptibility is also seen in young animals (<2 years) [20,52], such as those included in presented study. These data suggest that there is no significant difference in susceptibility between the sexes which could significantly influence our results and conclusions.

Interestingly, a predictive link between the swelling at the site of viral inoculation and the clinical outcome was seen. Following the initial swelling after the intradermal injection, two different evolutions of the swellings could be observed. At 5 dpi, the local swelling either stabilized in size (or even started to decrease) or continued to increase. The former was linked to a mild or unapparent clinical picture whereby the animals did not develop nodules. In contrast, when the local swelling continued to increase in size after 5 dpi, the animals developed noduli. The difference between the animals developing skin lesions or not became significant from 7 dpi onwards. This phenomenon was already described by Weiss in 1968 [58] and similar findings, albeit on a limited number of animals, were reported by Sanz-Bernardo et al. (2020) [43]. This indicates that the observation is not strain- or laboratory-dependent. As the difference in the development of the local swelling after intradermal challenge between vaccinated animals without skin lesions and control animals with skin lesions was also significant from 7 dpi, this observation can provide a useful tool for in vivo experimental LSDV studies and vaccine quality control.

## 5. Conclusions

All evaluated vaccines proved to be highly efficient in protecting the animals against a viral challenge under standardized conditions. These data support the protective effect seen in the field with live attenuated LSD-based vaccines. The vaccine should be fit for purpose for the region where it will be used, and the final vaccine choice will depend on the combined outcome of the vaccine efficacy, safety and the independent batch control including verification of the vaccine virus identity. The animal challenge model and the in vitro methods used proved adequate and robust for the comparison of LSDV-based vaccines. This model will further be used to evaluate and compare sheeppox- and goatpox-based vaccines against LSD.

## Figures and Tables

**Table 1:** Clinical scoring table. (a) The averaged diameter of the 4 intradermal inoculation sites in the neck; (b) Mild illness/Unwell: reduced responsiveness to stimuli, depression, reduced movement; Severe illness/Severely unwell: complete unresponsiveness, no movement (or laying down).

Body Temperature				Swelling at the Site of Challenge (Intradermal)		
1 Day		Consecutive Days			Diameter (a)	Points
°C	points	°C	points	Slightly enlarged	<2 cm	0.5
Up to 39.3	0.5	Up to 39.1	0	Moderately enlarged	2–4 cm	1
39.4 to 39.7		39.2 to 39.5	0.5	Large	4–7 cm	1.5
>39.8	1	39.6 to 40	1	Very large	>7 cm	2
		>40	1.5	General health status (b)		
Prescapular lymph node swelling				Normal		0
Enlarged			0.5	Mild illness/Unwell		1
Food intake				Severe illness/Severely unwell		2
Normal feeding			0	Number of noduli		
Slight decreased feeding			0.5	No nodules		0
Decreased feeding			1	Less than 10		1
Does not eat			1.5	More than 10		2
Nodule dissemination						
No noduli			0			
Local			1			
Generalized			2			

**Table 2:** Real-time PCR results of organ/tissue samples taken at necropsy form the control animals. The number of positive samples is expressed as percentages. Clinical: animals which developed typical LSDV skin lesions. Non-Clinical: animals without typical LSDV skin lesions.

Organ/Tissue Type	Overall	Clinical	Non-Clinical	Difference
Inoculation site	93.3	100	88.9	11.1
Normal skin	73.3	83.3	66.7	16.7
Nasal mucosa	53.3	100	22.2	77.8
Skin lesions	100	100	/	/
Inguinal Lnn	40	100	0	100
Prescapular Lnn	40	83.3	11.1	72.2
Submandibular Lnn	53.3	100	22.2	77.8
Bronchial Lnn	20	50	0	50
Mesenteric Lnn	20	50	0	50
Mediastinal Lnn	33.3	66.7	11.1	55.6
Tonsils	26.7	50	11.1	38.9
Iliacal Lnn	26.7	66.7	0	66.7
Parotid	60	100	0	100
Lung	33.3	66.7	11.1	55.6
Liver	13.3	16.7	11.1	5.6
Spleen	20	50	0	50
Rumen	53.3	83.3	33.3	50
Kidney	20	50	0	50
Tongue	40	83.3	11.1	72.2
Testis	26.7	66.7	0.0	66.7
Epididymis	40	66.7	22.2	44.4
Masseter	60	100	0	100
Musculus trapezius	60	100	0	100
Musculus psoas major	40	66.7	0	66.7
Quadriceps	60	100	0	100

**Table 3:** The summarized fever response after vaccination (dpv) in the vaccinated groups. N: number of animals with elevated body temperatures; Tmax: highest body temperature measured (in °C) in the group; Dmax: longest period of elevated body temperatures (in days) in the group; Damax: averaged longest consecutive period with elevated body temperatures (in days); Ntdays: total of days with elevated body temperatures; Nadays: average number of elevated body temperatures (in days); between brackets: the number of days with body temperature higher than 39.4 °C.

	OBP	Lumpyvax	Kenyavac	Herbivac	MCI
N	6	7	5	4	4
Tmax	40.5	40.6	40.8	40	40.4
Dmax	5	7	7	4	6
Damax	2.5	4	4.4	2.25	3.25
Ntdays	25 (21)	72 (42)	34 (26)	12 (12)	15 (15)
Nadays	4.2 (3)	10.3 (6)	6.8 (3.7)	3 (3)	3.8 (3.8)

## Data Availability

The main data presented in this study are available within the study itself and other data may be made available through contact with the corresponding author.

## Supplementary Materials

The following are available online at https://www.mdpi.com/article/10.3390/vaccines9050473/s1, Figure S1: Corrected OD values post-vaccination for OBP (A), Lumpyvax (B), Kenyavac (C), Herbivac (D) and MCI (E). Red line: positivity cut-off; light green line: weak positive cut-off; dark green line: medium positive cut-off.
