# Supplementary material for: Comparative Evaluation of Lumpy Skin Disease Virus-Based Live Attenuated Vaccines
Source: Vaccines (Basel). 2021 May 8;9(5):473. doi: 10.3390/vaccines9050473 (PMC8151199; doi:10.3390/vaccines9050473)
Supplement: Supplementary file 1 [file vaccines-09-00473-s001.zip › vaccines-1186525-SI.pdf]

Supplemental Figure S1

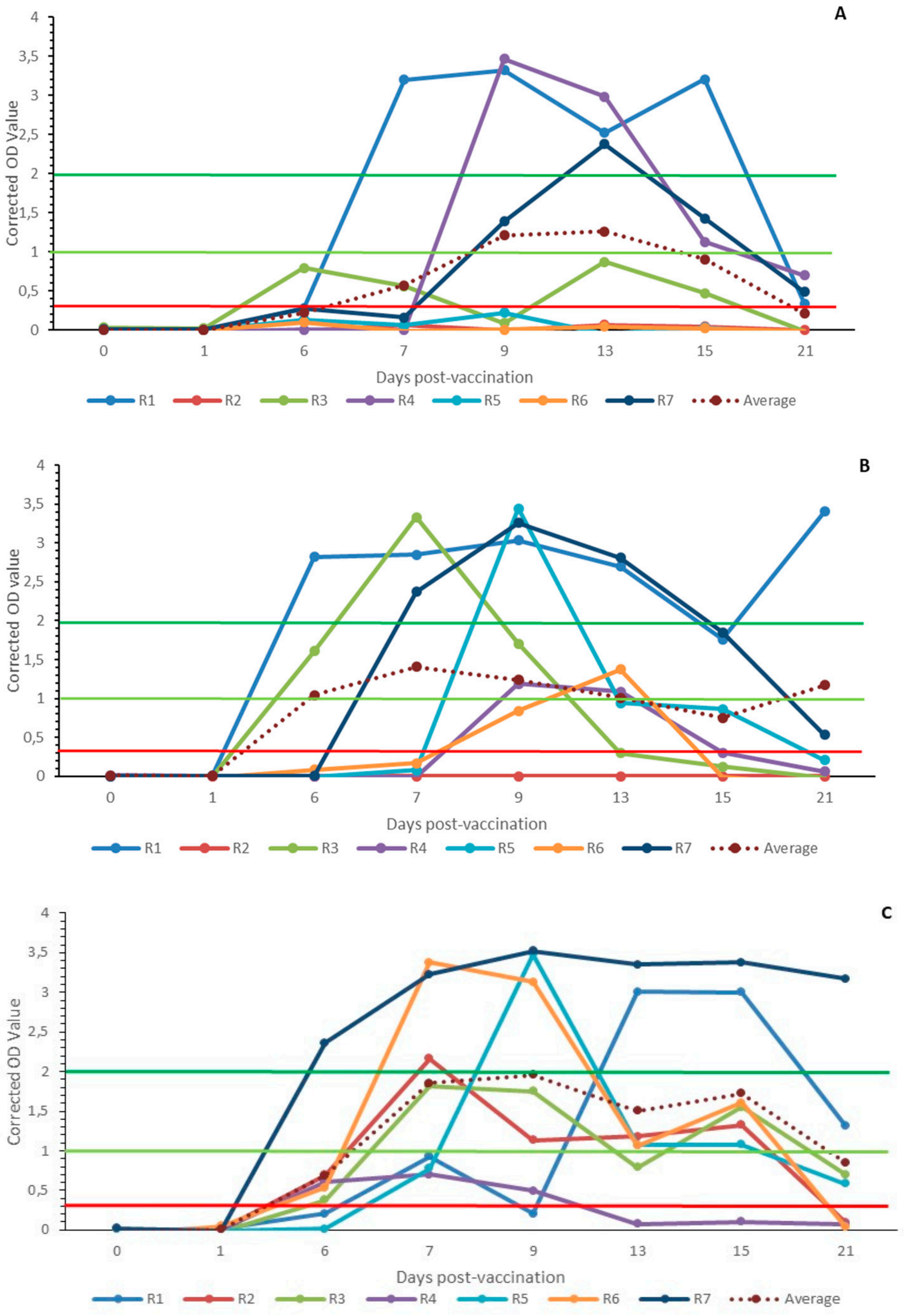

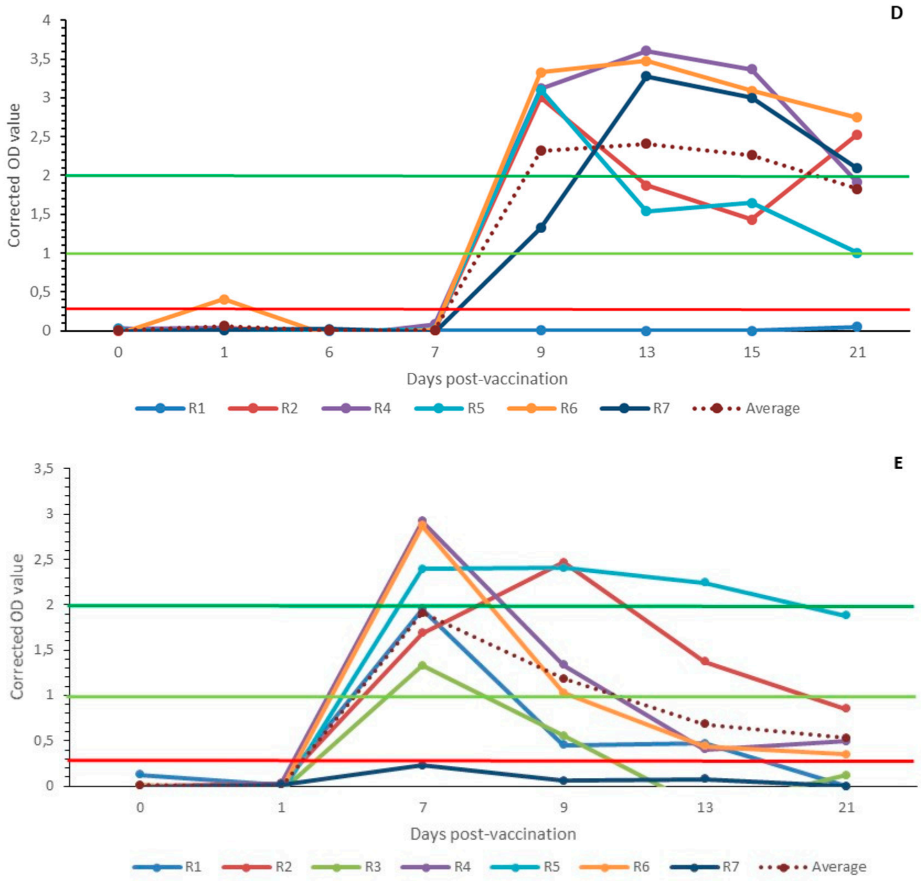

Figure S1: Corrected OD values post-vaccination for OBP (A), Lumpyvax (B), Kenyavac (C), Herbivac (D) and MCI (E). Red line: positivity cut-off; light green line: weak positive cut-off; dark green line: medium positive cut-off
